# Migration Behavior of ^137^Cs, ^79^Se, and ^99^Tc in Clay Rocks: Role of Competitive Adsorption Under Coexistence Conditions

**DOI:** 10.3390/ma19132835

**Published:** 2026-07-02

**Authors:** Yunfeng Shi, Song Yang, Hanhan Liu, Zhou Li, Longjiang Wang, Jun Tan, Weijie Chen, Ting Wang, Aiming Zhang, Bing Lian

**Affiliations:** 1 Department of Nuclear Environmental Science, China Institute for Radiation Protection (CIRP), Taiyuan 030006, China; syf541006935@126.com (Y.S.); yangsong@cirp.org.cn (S.Y.); lizhou@cirp.org.cn (Z.L.); wanglongjiang@cirp.org.cn (L.W.); thutanjun@163.com (J.T.); ae198cirp@163.com (W.C.); ting880218@163.com (T.W.); 18511558388@163.com (A.Z.); 2 Jiangsu Nuclear Power Corporation, Lianyungang 222042, China; liuhh02@cnnp.com

**Keywords:** competitive adsorption, clay rocks, radioactive isotopes, numerical modeling, anion repulsion

## Abstract

To address the issue of radioactive waste generated by the large-scale promotion and use of nuclear energy, safety evaluations of disposal sites in various surrounding rocks are essential. These evaluations are a prerequisite for ensuring the long-term safe disposal of radioactive waste. This study focuses on the blocking capacity of clay rocks concerning the advection–dispersion behavior of representative radionuclides such as ^137^Cs, ^79^Se, and ^99^Tc. It further examines the effects of competitive adsorption that arise when these three radionuclides coexist. (Since ^79^Se is difficult to obtain, ^75^Se was used as a substitute nuclide. In the mixed-nuclide experiments, the stable isotope Re was used to replace ^99^Tc.) The experimental findings revealed that competitive adsorption can significantly reduce the adsorption capability of clay rocks for ^137^Cs and ^79^Se, altering the adsorption mechanism. During the advection–dispersion process, the weak adsorption sites of ^137^Cs and ^79^Se on clay rocks become active after the strong adsorption sites are preferentially occupied, resulting in a decline in both adsorption quantity and rate. In the case of ^99^Tc, competitive adsorption weakens the effect of anion repulsion, leading to a reduction in the immobile liquid regions (*θ_im_*).

## 1. Introduction

Nuclear energy, as a safe, stable, efficient, and widely applicable low-carbon energy source, is considered a crucial element in addressing global climate change and advancing the transition to a clean and low-carbon energy structure [1]. By the end of June 2025, there were a total of 416 nuclear power units operational worldwide, with a combined installed capacity of approximately 380 million kilowatts [2]. The significant scale of the nuclear power industry inevitably results in a substantial increase in radioactive waste. Currently, the internationally accepted method for disposing of radioactive waste is geological disposal. Radioactive waste is categorized into six groups based on radioactivity levels, including high-level, medium-level, low-level, extremely low-level, extremely short-lived, and exempted waste. High-level, medium-level, low-level, and extremely low-level radioactive waste require separate disposal in deep geological, medium-depth, and near-surface facilities for long-term isolation. A “multiple barrier system” must be used to prevent adverse environmental impacts during the safety evaluation period [3]. The surrounding rock at the disposal site serves as the final defense in the “multiple barrier system,” effectively preventing nuclide leakage through its low permeability, nuclide adsorption, and other characteristics. Owing to variations in the physical and chemical properties of different rock types, countries have extensively researched various surrounding rock materials and established evaluation criteria for disposal site surrounding rock [4]. Clay rock has emerged as a prominent bedrock choice for radioactive waste disposal sites due to its low permeability, strong nuclide adsorption capabilities, and effective fracture self-healing ability [5]. Scholars have conducted thorough investigations into the ability of clay rocks to block various nuclides. For instance, Zheng et al. studied the advection–dispersion behavior of U(VI) in clayrocks, identifying montmorillonite minerals as the primary adsorption agents and noting the significant impact of bicarbonate on U(VI) migration rates. They developed a migration numerical model incorporating THMC four-field coupling [6]. Hossain et al. compared the advection–dispersion behavior of ^60^Co and ^137^Cs in clay rocks of different particle sizes, highlighting the substantial influence of particle size on the ability of clay rocks to block nuclide migration [7]. Recent studies by our research group have focused on the migration behavior of various nuclides in clay rocks. For example, in 2025, our findings indicated that the advection–dispersion behavior of ^226^Ra aligns with the non-equilibrium two-region model, revealing mobile and immobile zones during the migration process of ^226^Ra, influenced by two adsorption mechanisms [8]. Similarly, experiments on the advection–dispersion of the anionic nuclide ^99^Tc in clay rocks demonstrated that surface anions on clay rocks induce an anionic repulsion effect on ^99^Tc, significantly accelerating nuclide migration [9].

Summarizing relevant research results, it is noted that clay rocks exhibit poor permeability and robust self-healing capabilities. Consequently, scholarly attention is predominantly directed toward the diffusion patterns of nuclides prior to their ingress into the aquifer, leading to a relatively limited exploration of advection–dispersion phenomena. Moreover, the design and modeling of advection–dispersion migration experiments have overlooked scenarios involving multi-nuclide leakage and release. Consequently, the migration behavior parameters derived are primarily suited for evaluating the barrier efficacy of adjacent rocks against a singular nuclide. This disparity from real-world scenarios hampers the efficacy of safety assessments for disposal sites.

The main impact of the coexistence of multiple nuclides is the emergence of competitive adsorption phenomena among different nuclides. With an increase in the types and quantities of coexisting nuclides, this competitive adsorption phenomenon intensifies, leading to a notable reduction in the adsorption capacity of solid-phase media for multiple nuclides compared to when a single nuclide is present [10,11]. While limited research exists on the impact of competitive adsorption on the advection–dispersion behavior of radioactive nuclides, numerous studies have explored this phenomenon in non-radioactive fields, such as modeling the migration of heavy metal ions in soil. Padilla et al. compared the advection–dispersion behavior of Ni(II) and Zn(II) under single charge and mixed coexistence [12]. They found that the competitive adsorption of Ni(II) and Zn(II) ions can diminish the adsorption blocking effect of soil and accelerate solute migration. Li et al. observed that Cd migrates more rapidly in mixed solutes containing Pb [13]. They developed a solute transport mathematical model that accounts for competitive adsorption. Cao et al. conducted a systematic examination of the occurrence and migration behavior of As, Cd, and Cr in various typical soil types in China [14]. They highlighted that the coexistence of heavy metal ions can influence their migration within soils.

The radioactive waste disposal site involves more than 200 types of nuclides [15], which can be categorized based on their adsorption capacity into three groups: strongly adsorbed nuclides (e.g., ^137^Cs, ^239^Pu, etc.); weakly adsorbed nuclides (e.g., ^79^Se and ^90^Sr); and non-adsorbed nuclides (e.g., ^99^Tc, ^129^I, etc.). ^137^Cs (half-life of 30.2 years) is a significant nuclide in waste from nuclear power plants, posing risks to human health through ecosystems and food chains due to its high bioavailability. Its Cs^+^ chemical form allows strong adsorption onto solid surfaces through ion exchange with most media, as demonstrated by its strong adsorption properties [16]. However, recent studies indicate that seawater can notably enhance Cs migration in soil [17]. ^79^Se (half-life of 3.27 × 10^5^ years) is a crucial migration fission product in the high-level radioactive waste disposal process [18]. The migration behavior of ^79^Se highlights distinct differences based on the valence state of the nuclides. Previous research has shown that Se(VI) exhibits easier migration compared to Se(IV) [19]. ^99^Tc (half-life of 2.14 × 10^5^ years) is another important nuclide in nuclear power plant waste, with a high fission yield in reactors. Given that ^99^Tc commonly exists in water as “TcO_4_^−^,” its migration is affected by “anion repulsion” and accelerates nuclide migration [9]. While extensive research has been conducted on the adsorption capacity of nuclides under multiple coexisting nuclides competitive adsorption, much less attention has been paid to their diffusion and advection–dispersion behavior. Scholars worldwide have extensively studied the migration behavior of typical representative nuclides in various media. However, further research is necessary to assess the applicability of these findings in systems with multiple coexisting nuclides. Moreover, existing work lacks a systematic framework that spans from nuclide selection through phenomenological analysis to numerical simulation.

In this study, physical and numerical simulation experiments on the adsorption and advection–dispersion behavior of ^137^Cs, ^79^Se, and ^99^Tc in clay rocks were conducted. The migration behavior of individual nuclides in clay rocks was compared, with particular emphasis on the influence of competitive adsorption on migration-blocking performance under both single-occurrence and coexistence conditions. Various numerical models were employed to determine essential characterization parameters, thereby supporting the safety assessment of radioactive waste disposal sites.

## 2. Adsorption and Advection–Dispersion Mechanisms

### 2.1. Thermodynamic Adsorption Model

Thermodynamic adsorption model is utilized to depict the distribution of nuclides in the solid and liquid phases upon reaching adsorption equilibrium. It enables the analysis of nuclide adsorption types by assessing the suitability of various models [20]. The models examined in this study comprise:

(1) Distribution coefficient model

The distribution coefficient is a parameter that characterizes the distribution of nuclides in the solid-liquid phase, determined by the ratio of nuclide activity concentration in the solid phase to that in the liquid phase. The formula is [21]:

(1) $$K_{d}=\frac{(c_{o}-c_{e})V}{c_{e}m}$$

where $c_{o}$ is the initial activity concentration of nuclides in the liquid phase ($\mathrm{Bq}\cdot {\mathrm{dm}}^{-3}$), $c_{e}$ is the activity concentration of nuclides in the liquid phase at adsorption equilibrium ($\mathrm{Bq}\cdot {\mathrm{dm}}^{-3}$), *V* is the total volume of the solution (${\mathrm{dm}}^{3})$, and *M* is the mass of the solid phase medium (kg).

(2) Langmuir adsorption model (abbreviated as L-M)

This model assumes that the solid-phase medium functions as a single-layer surface adsorption, with uniform adsorption sites and independent adsorption of nuclides. The formula is [20]:

(2) $$\frac{C_{e}}{q_{e}}=\frac{C_{e}}{q_{\max}}+\frac{1}{q_{\max}k_{l}}$$

where $q_{\max}$ is the maximum adsorption capacity of the solid-phase medium (Bq∙kg^−1^) and $c_{e}$ is the concentration of nuclide activity in the liquid phase at adsorption equilibrium ($\mathrm{Bq}\cdot {\mathrm{dm}}^{-3}$). $k_{l}$ is a constant related to the heat of adsorption, which can determine whether the adsorption process is a chemical or physical process (${\mathrm{dm}}^{3}\cdot {\mathrm{Bq}}^{-1}$), and $q_{e}$ is the adsorption capacity of the solid-phase medium for nuclides at the average adsorption time (Bq∙kg^−1^).

(3) Freundlich adsorption model (abbreviated as F-M)

This model assumes that the surface position of the adsorbent has different adsorption sites, and the formula is [20,22]:

(3) $$lnq_{e}=lnk_{f}+\frac{1}{n}lnC_{e}$$

where $k_{f}$ is a constant denoting adsorption capacity; *n* represents the adsorption strength when the equilibrium concentration of metal ions is 1, with a range of 1 < *n* < 10; and $c_{e}$ is the activity concentration of nuclides in the liquid phase at adsorption equilibrium ($\mathrm{Bq}\cdot {\mathrm{dm}}^{-3}$).

(4) Langmuir–Freundlich adsorption model (abbreviated as L-F)

This model couples the Langmuir isotherm adsorption model with the Freundlich isotherm adsorption model and introduces dimensionless parameters to determine whether the adsorption site is a single site [23,24]. The expression formula is:

(4) $$q_{e}=\frac{q_{\max}{\left(k_{l}C_{e}\right)}^{\beta }}{1+{\left(k_{l}C_{e}\right)}^{\beta }}$$

where β (0 < β < 1) characterizes the degree of non-uniformity of adsorption sites on the surface of solid-phase media, with a smaller value indicating a higher degree of non-uniformity.

### 2.2. Adsorption Kinetics Adsorption Model

This type of model is used to describe the changes in the adsorption capacity of nuclides over time. Moreover, it can analyze the types of nuclide adsorption based on the applicability of different models [20]. The models involved in this study include:

(1) Pseudo-first-order model (abbreviated as P-F)

This model suggests that the reaction rate constant at the interface between nuclides and solid-phase media can be described by a quasi-first-order kinetic model, with the formula:

(5) $$ln\left(q_{e}-q_{t}\right)=lnq_{e}-k_{1}t$$

where $q_{e}$ is the adsorption capacity of the solid phase medium for nuclides at adsorption equilibrium (Bq∙g^−1^), $k_{1}$ is the first-order adsorption rate constant (d^−1^), $q_{t}$ is the adsorption amount of the solid phase medium on the nuclide at time *t* (Bq∙g^−1^), and *t* is the time (d).

(2) Pseudo-second-order model (abbreviated as P-S)

The model assumes the solid-phase medium functions as a single-layer surface adsorption, with uniform adsorption sites and independent adsorbed nuclides. The formula is:

(6) $$\frac{t}{q_{t}}=\frac{1}{k_{2}.{q_{e}}^{2}}+\frac{t}{q_{e}}$$

where $q_{e}$ is the adsorption capacity of the solid-phase medium for nuclides at adsorption equilibrium (Bq∙kg^−1^), $k_{2}$ is the second-order adsorption rate constant (kg∙Bq^−1^∙d^−1^), $q_{t}$ is the adsorption capacity of the solid-phase medium for the nuclide at time *t* (Bq∙kg^−1^), and *t* is the time (d).

(3) Multi-stage sorption kinetic model (abbreviated as M-S)

The model divides the solid-phase medium into various stages of adsorption rate potentials determined by adsorption rate. It characterizes the adsorption process by defining adsorption rate constants and desorption rate constants. The formula is [25]:

(7) $$\frac{\partial S_{i}}{\partial t}=k_{fi}c\left(1-\frac{s_{i}}{s_{mi}}\right)-k_{ri}s_{i}$$

(8) $$\frac{\partial c}{\partial t}=\frac{M}{V}\left[-\sum_{i}k_{fi}c\left(1-\frac{s_{i}}{s_{mi}}\right)+\sum_{i}k_{ri}s_{i}\right]$$

where c is the activity concentration of the nuclide in the liquid phase ($\mathrm{Bq}\cdot {\mathrm{cm}}^{-3}$), $S_{i}$ represents the adsorption capacity of the *i* (*i* = 1, 2, 3…) class adsorption sites on the surface of the solid-phase medium (Bq∙g^−1^), *t* is time, $k_{fi}$ is the adsorption rate constant for class *i* adsorption sites (${\mathrm{cm}}^{3}$∙(gd)^−1^), $k_{ri}$ is the desorption rate constant for class *i* adsorption sites (d^−1^), $s_{mi}$ represents the maximum adsorption capacity per unit of type *i* adsorption sites (Bq∙g^−1^), *M* is the mass of the solid-phase medium (g), and *V* is the volume of the liquid-phase medium (${\mathrm{cm}}^{3}$).

### 2.3. Advection–Dispersion Model

This model is utilized to characterize the convective dispersion migration behavior of nuclides in solid-phase media under the influence of water flow. Existing models are predominantly developed based on physical factors such as pore medium morphology and chemical factors such as adsorption during nuclide migration. The models considered in this study are:

(1) Equilibrium transport (abbreviated as E-T)

The one-dimensional equation is as follows [26]:

(9) $$\frac{\partial }{\partial t}(\theta c_{r}+\rho_{b}s)=\frac{\partial }{\partial x}(\theta D\frac{\partial c_{r}}{\partial x}-J_{w}c)-\theta \mu_{l}c_{r}-\rho_{b}\mu_{s}s+\theta r_{l}\left(x\right)+\rho_{b}r_{s}\left(x\right)$$

where $c_{r}$ is the volume-averaged or resident concentration of the liquid phase (ML^−3^), *s* is the concentration of the adsorbed phase (MM^−1^), *D* represents the dispersion coefficient (L^2^T^−1^), θ is the volumetric water content (L^3^L^−3^), $J_{w}$ is the volumetric water flux density (LT^−1^), $\rho_{b}$ is the soil bulk density (ML^−3^), $\mu_{l}$ and $\mu_{s}$ are the first-order decay coefficients for the degradation of the solute in the liquid and adsorbed phases, respectively (T^−1^), $r_{l}$(*ML*^−3^ *T*^−1^) and $r_{s}$ (MM^−1^ T^−1^) are the zero-order production terms for the liquid and adsorbed phases, respectively, *x* is the distance (L), and *t* is the time (T).

The solute adsorption by the solid phase is expressed using a linear isotherm as follows:

(10) $$s=K_{d}c_{r}$$

where $K_{d}$ is an empirical distribution constant (M^−1^L^3^). Using Equation (10) and assuming a steady-state flow in a homogeneous soil, Equation (9) may be rewritten as follows:

(11) $$R\frac{\partial c_{r}}{\partial t}=D\frac{\partial^{2}c_{r}}{\partial x^{2}}-v\frac{\partial c_{r}}{\partial x}-\mu c_{r}+r\left(x\right)$$

where v($=\frac{J_{w}}{\theta }$) is the average pore–water velocity. *R* is the retardation factor, defined as $R=1+\frac{\rho_{b}K_{d}}{\theta }.$ The terms μ and r denote the combined first- and zero-order rate coefficients, respectively, given by $\mu =\mu_{l}+\frac{\rho_{b}K_{d}\mu_{s}}{\theta }$ and $r(x)=r_{l}(x)+\frac{\rho_{br_{s}(x)}}{\theta }.$

(2) One/two-sites non-equilibrium transport (abbreviated as O/T-S)

If the time for adsorption and desorption equilibrium is insufficient, solute adsorption by the solid phase is characterized using non-equilibrium one- and two-site models. The non-equilibrium transport model with one/two sites is described by [27]:

(12) $$\left(1+\frac{f\rho_{b}K_{d}}{\theta }\right)\frac{\partial c}{\partial t}=D\frac{\partial^{2}c}{\partial x^{2}}-v\frac{\partial c}{\partial x}-\frac{\alpha \rho_{b}}{\theta }\left[\left(1-f\right)K_{d}c-s_{k}\right]-\mu_{l}c-\frac{f\rho_{b}K_{d}\mu_{s,e}c}{\theta }+r_{l}\left(x\right)+\frac{f\rho_{b}r_{s,e}\left(x\right)}{\theta }$$

(13) $$\frac{\partial s_{k}}{\partial t}=\alpha \left[\left(1-f\right)K_{d}c-s_{k}\right]-\mu_{s,k}+\left(1-f\right)r_{s,k}\left(x\right)$$

where α is first-order kinetic constant (T^−1^), f is the proportion of equilibrium points at all adsorption points, and *e* and *k* refer to the adsorption equilibrium and adsorption kinetics points, respectively. When *f* = 0, the two-sites model becomes a one-site model.

(3) Two-region non-equilibrium transport (abbreviated as T-T)

A two-region transport model was proposed, assuming that the liquid phase can be divided into mobile (flowing) and immobile (stagnant) regions, which result from anion exclusion. The exchange of solutes between these liquid regions is modeled as a first-order kinetic process. The two-region solute transport model can be represented as [28]:

(14) $${(\theta }_{m}+f\rho_{b}K_{d})\frac{\partial c_{m}}{\partial t}=\theta_{m}D_{m}\frac{\partial^{2}c_{m}}{\partial x^{2}}-J_{w}\frac{\partial c_{m}}{\partial x}-a\left(c_{m}-c_{im}\right)-\left(\theta_{m}\mu_{l,m}+f\rho_{b}K_{d}\mu_{s,m}\right)c_{m}+\theta_{m}r_{l,m}\left(x\right)+f\rho_{b}r_{s,m}(x)$$

(15) $${(\theta }_{im}+\left(1-f\right)\rho_{b}K_{d})\frac{\partial c_{im}}{\partial t}=a\left(c_{m}-c_{im}\right)-\left(\theta_{im}\mu_{l,im}+\left(1-f\right)\rho_{b}K_{d}\mu_{s,im}\right)c_{im}+\theta_{im}r_{l,im}\left(x\right)+\left(1-f\right)\rho_{b}r_{s,im}\left(x\right)$$

where the subscripts *m* and *im* refer to the mobile and immobile liquid regions, respectively; $J_{w}=v\times \theta =v_{m}\times \theta_{m}$ is the volumetric water flux density; f represents the fraction of adsorption sites that equilibrates with the mobile liquid phase; and a is the first-order mass transfer coefficient governing the rate of solute exchange between the mobile and immobile liquid regions. Furthermore, θ is equal to $\theta_{m}+\theta_{im}$, $\mu_{l,m}$ and $\mu_{l,im}$ are first-order decay coefficients for the mobile and immobile liquid phases, $\mu_{s,m}$ and $\mu_{s,im}$ are first-order decay coefficients for the mobile and immobile adsorbed phases, $r_{l,m}$ and $r_{l,im}$ are zero-order production for the mobile and immobile liquid phases, and $r_{s,m}$.and $r_{s,im}$ are zero-order production terms for the mobile and immobile adsorbed phases, respectively.

## 3. Experiment

### 3.1. Clay Rocks

**Figure 1:**
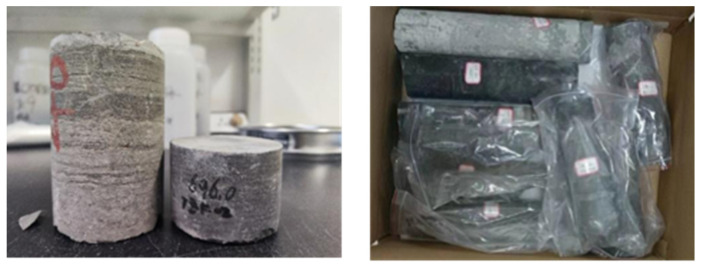
Sample display (clay rocks, collected from Inner Mongolia in northern China).

The samples were collected from Inner Mongolia in northern China, as shown in Figure 1. Following collection, the samples were crushed, washed three times with deionized water, and dried at 100 ± 10 °C for 24 h before being set aside. Analysis included determining the mineral composition, chemical element composition, cation exchange capacity, and organic matter content of the rock samples.

### 3.2. Adsorption Experiment

Batch experiments were conducted in the single-nuclide adsorption laboratory using the radioactive nuclides ^137^Cs (China Tongfu Co., Ltd., Nantong, China) and ^79^Se (later replaced by ^75^Se (China Tongfu Co., Ltd., Nantong, China) and ^99^Tc (China Tongfu Co., Ltd., Nantong, China). The multi-nuclide coexistence adsorption test involved stable isotopes Cs (Tianjin Kermel Chemical Reagent Co., Ltd., Tianjin, China), Se (Shanghai Aladdin Biochemical Technology Co., Ltd., Shanghai, China), and Tc (later replaced by Re (Sigma-Aldrich Corporation, Burlington, MA, USA). In total, 1 g of pre-treated clay rock sample (<75 μm) was placed into a 15 mL pre-treated centrifuge tube. Subsequently, 10.0 mL of a mixed solution of nuclides and ultrapure water was added, with a solid–liquid ratio of 1:10 g/mL and a pH value ranging from 7.3 to 8.0 and Eh value ranging from 0.60 V to 0.75 V. The mixture was continuously shaken at 25 °C to ensure complete contact between the solid and liquid phases. Once the solute had reached the designated time in both the aqueous and solid phases, the solid-liquid mixture was separated using a centrifuge (L550, Hunan Xiangyi Laboratory Instrument Development Co., Ltd., Changsha, China). The nuclide content in the water and soil samples was measured, and the activity concentration was calculated. The parameters and measurement methods for each solute in the adsorption test are detailed in Table 1 and Table 2.

All samples analyzed in this study were water samples. Radioactive nuclides were measured using an ultra-low background liquid scintillation spectrometer (LSA3000, Shanghai Xinman Sensing Technology Co., Ltd., Shanghai, China) and a high-purity germanium gamma spectrometer (GEM, AMETEK ORTEC, Oak Ridge, TN, USA). Stable element analyses were performed using an atomic absorption spectrometer (240FS AA, Agilent Technologies, Santa Clara, CA, USA) and an inductively coupled plasma spectrometer (Plasma 1500, Jiangsu Institute of Testing Technology Co., Ltd., Yangzhou, China).

### 3.3. Column Experiment

**Figure 2:**
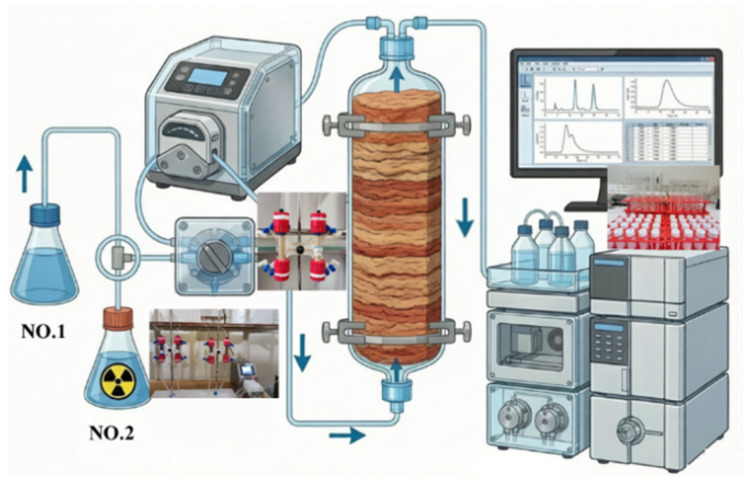
Schematic diagram of experimental setup (comprises three main sections: the injection section, column section, and collection section).

The experimental setup comprises three main sections: the injection section, column section, and collection section. The injection section involves bottle 1 (containing ultrapure water) and bottle 2 (containing the solute), connected to a peristaltic pump via a controlled three-way valve to facilitate solute injection. The column is filled with clay rock samples, and filters are installed at both the upper and lower ends to prevent sample loss. The collection section is designated for gathering the effluent from the column, and analysis is conducted after completing sample collection at regular intervals, as illustrated in Figure 2.

The pre-treated clay rock (particle size 250–350 μm) was filled with a density of 1.25 g/cm^3^ into the glass column (length: 10 cm; diameter: 1.6 cm). The three-way valve was adjusted, the peristaltic pump was opened, and ultrapure water was injected from bottle 1 into the column from the bottom to complete the saturation treatment of the sample water (the flow rate was 0.25 cm^3^·min^−1^). Subsequently, the HTO solution was injected from bottle 2, the three-way valve was adjusted, and the peristaltic pump was opened to instantly introduce HTO into the soil column (the injected pulse volume was 0.1 cm^3^; the injection time was 1 s). The samples were collected at regular intervals at the outflow end and analyzed them (repeat the experiment three times). After rinsing the HTO from bottle 2, ^137^Cs, ^75^Se (instead of ^79^Se), ^99^Tc, and mixed solutions of Cs, Se, and Re (instead of ^99^Tc) were sequentially injected into the soil column in an instantaneous manner. The samples were collected and analyzed at regular intervals at the outflow end. Owing to the slow migration rate of ^137^Cs in clay rock under a single-nuclide system, which cannot penetrate the soil, test samples were obtained by disassembling the soil column. In the numerical model, the treatment of boundary conditions is instantaneous injection, which is consistent with the experimental process. The details of the samples are provided in Table 2.

### 3.4. Numerical Simulation and Parameter Evaluation

The data from adsorption experiments and column experiments were analyzed using MATLAB Version R2022b (MathWorks, Natick, MA, USA) (lsqcurvefit) and STANMOD (STudio of ANalytic MODels) Version 2.08 (U.S. Salinity Laboratory, Riverside, CA, USA), respectively. STANMOD is a Windows-based computer software package utilized for assessing solute transport in porous media through analytical solutions of the advection–dispersion solute transport equation. This software incorporates revised editions of the CXTFIT code for determining solute transport parameters via nonlinear least squares parameter optimization techniques. Both software applications utilize the root mean square error (RMSE) to quantify the goodness of fit, defined as

(16) $$\mathrm{RMSE}=\sqrt{\frac{\sum_{i}^{N}{\left(C_{p}-C_{e}\right)}^{2}}{N}}$$

where $C_{e}$ is the experimental result, the deviation of $C_{p}$ from the predicted value and the quantity of *N* experimental data.

## 4. Results and Discussion

### 4.1. Analysis of Minerals and Chemical Composition

**Figure 3:**
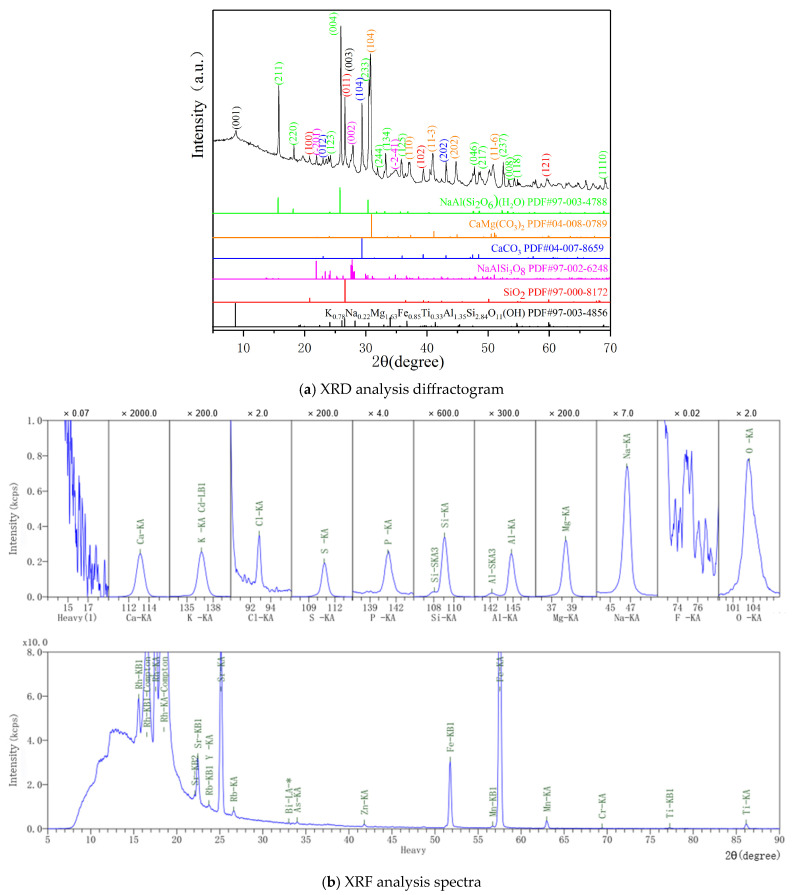
Analysis results (XRD characterizes the phases present in the clay rocks, while XRF reflects their chemical composition). The asterisk marks the spectral overlap interference between Bi-Lα and As-Kα, which requires peak fitting correction during quantitative analysis.

XRD and XRF analysis techniques were utilized to examine the mineral and chemical composition of clay rocks. X-ray diffraction (XRD) was performed on a Rigaku SmartLab SE diffractometer (Rigaku, Tokyo, Japan) using Cu Kα radiation, operated at 40 kV and 40 mA. The black curve represents the measured diffraction spectrum, and the colored lines correspond to standard PDF cards of each identified mineral phase. The numbers marked above diffraction peaks are the corresponding crystal plane indices (hkl); X-ray fluorescence (XRF) analysis was carried out on a Thermo Fisher PFX-480 X-ray fluorescence spectrometer (Thermo Fisher Scientific, Waltham, MA, USA) operated at 60 kV, the sample had no plastic covering film, and the X-ray beam path was under vacuum mode. The collimator mask was 29 mm; only elements with a concentration greater than 10 ppm and a mass fraction exceeding the estimated error were reported. The pertinent findings are presented in Figure 3 and Table 3. Following the analysis, the primary mineral constituents identified include dolomite, zeolite, plagioclase, and mica, while the principal chemical components consist of SiO_2_, CaO, MgO, and Al_2_O_3_. The cation exchange capacity is 10.936 cmol∙kg^−1^ (Chinese Standard Method: Hexamminecobalt(III) Chloride Extraction-Spectrophotometry (HJ 889-2017) [29]), and the organic matter content is 27.163 g∙kg^−1^.

### 4.2. Adsorption Experiment Results

**Figure 4:**
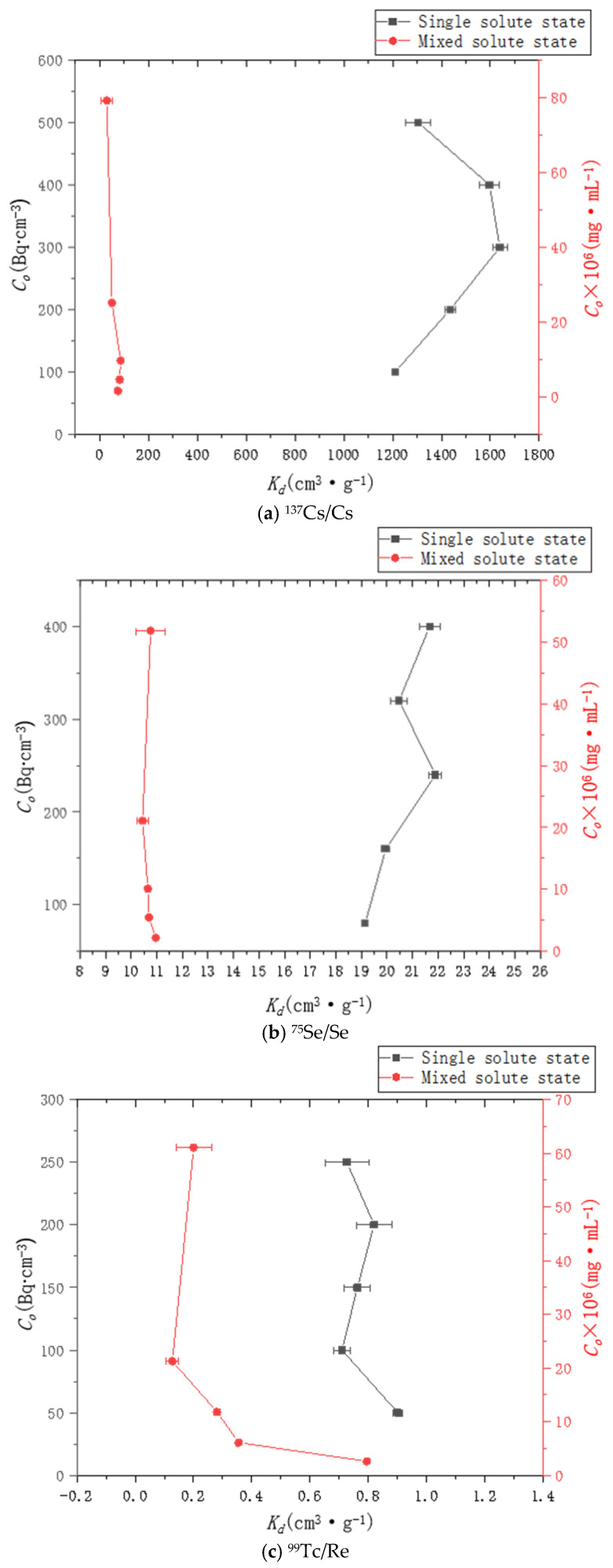
Adsorption behavior (the adsorption of ^137^Cs, ^75^Se, and ^99^Tc in a single-nuclide system, as well as Cs, Se, and Re in a mixed-nuclide system in clay rocks under various initial activity concentrations, was determined through batch experiments).

The adsorption of ^137^Cs, ^75^Se, and ^99^Tc in a single-nuclide system, as well as Cs, Se, and Re in a mixed-nuclide system in clay rock under various initial activity concentrations, was determined through batch experiments, as illustrated in Figure 4. Overall, whether in a single-nuclide system or a mixed-nuclide system, the adsorption capacity of clay rock for the three nuclides follows the order ^137^Cs/Cs > ^75^Se/Se > ^99^Tc/Re. Notably, the distribution coefficient of ^99^Tc/Re is relatively low. The calculation of the solid-phase adsorption capacity in the experiment involved subtracting the liquid-phase content from the total solute input, accounting for potential measurement errors in the liquid-phase sample and solute loss during operations. Consequently, it is inferred that clay rock exhibited insignificant adsorption of ^99^Tc/Re in this experiment. In addition, the experimental data clearly indicate that the presence of a mixed-nuclide system can notably diminish the adsorption capacity of clay rocks for ^137^Cs and ^79^Se, suggesting competitive adsorption.

**Figure 5:**
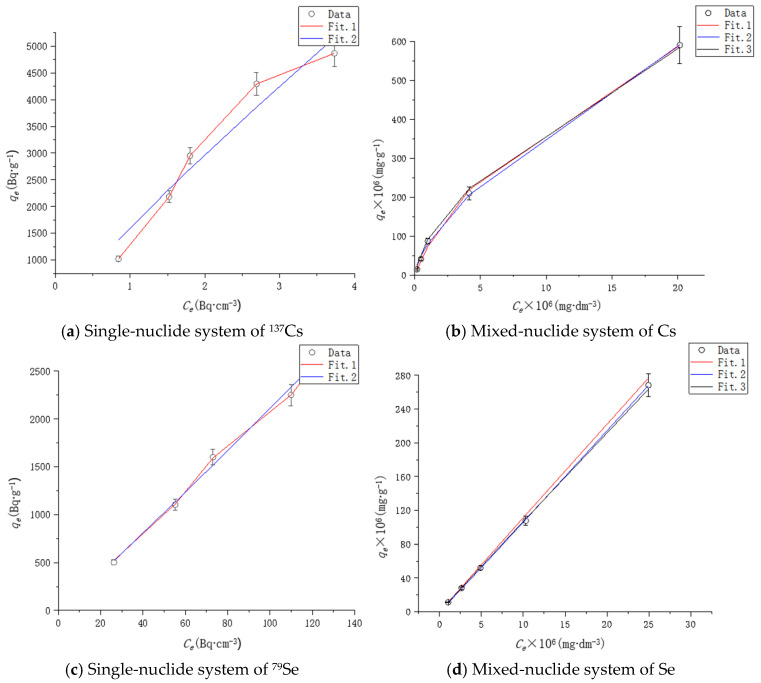
Adsorption thermodynamic curve (Fit. 1 represents the fitting result of the Langmuir adsorption model (L-M), Fit. 2 represents the fitting result of the Freundlich adsorption model (F-M), and Fit. 3 represents the fitting result of the Langmuir–Freundlich adsorption model (L-F)).

**Figure 6:**
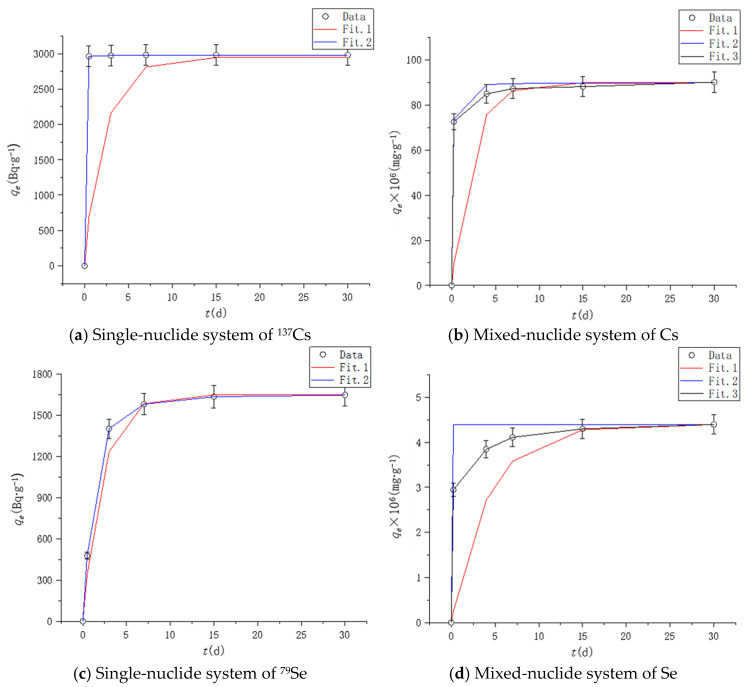
Adsorption kinetics curve (Fit. 1 represents the fitting result of the pseudo-first-order model (P-F), Fit. 2 represents the fitting result of the pseudo-second-order model (P-S), and Fit. 3 represents the fitting result of the multi-stage sorption kinetic model (M-S)).

To further analyze the impact of competitive adsorption on the adsorption capacity of ^137^Cs and ^79^Se, various numerical models were utilized to fit the thermodynamic and kinetic curves of clay rock adsorption of ^137^Cs and ^79^Se. The outcomes are presented in Figure 5 and Figure 6 and Table 4 and Table 5. The adsorption thermodynamic and kinetic curves of ^137^Cs and ^79^Se in a single-nuclide system can be more accurately described using the Langmuir model and quasi-second-order kinetic model, respectively. By combining the assumptions of these models, it is evident that the adsorption of ^137^Cs and ^79^Se by clay rock in a single-nuclide system conforms to single-layer surface adsorption, with uniform adsorption sites and independent adsorbed nuclides. For the adsorption behavior of Cs and Se in a mixed-nuclide system, the Langmuir–Freundlich model and multilevel kinetics (second order) are required to effectively fit the thermodynamic and kinetic curves, respectively. This suggests that, in the presence of multiple nuclides, the competitive adsorption of Cs and Se significantly alters the adsorption mechanism of clay rocks for ^137^Cs and ^79^Se by occupying relevant adsorption sites. As a result, the original single-site adsorption is transformed into a multi-site joint adsorption site. Thermodynamically, the adsorption sites can be categorized into “Langmuir model sites” and “Freundlich model sites” collectively, while in terms of kinetics, it is observed as the combined action of “fast adsorption sites” and “slow adsorption sites.”

### 4.3. Column Experiment Results

(1) Advection–dispersion experiment of HTO

**Figure 7:**
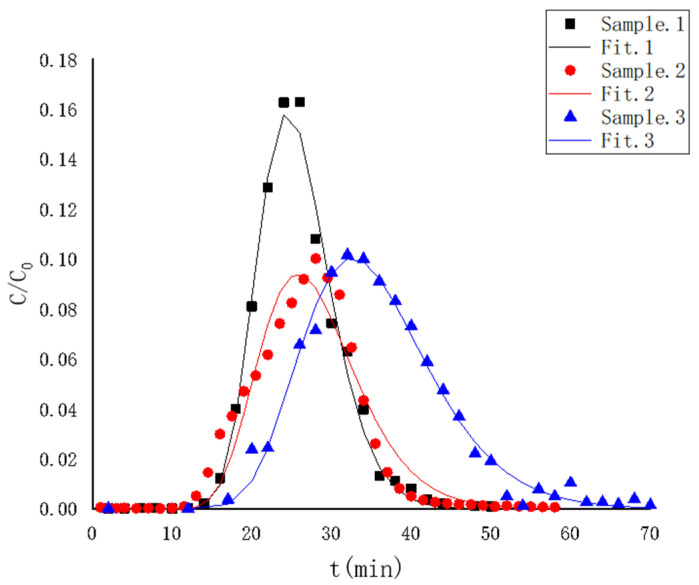
Advection–dispersion curve fitting of HTO.

Before fitting the advection–dispersion migration curves of ^137^Cs/Cs, ^75^Se/Se, and ^99^Tc/Re in clay rocks under single-nuclide and mixed-nuclide systems, it is essential to simulate the migration curves of HTO in various media to derive pertinent physical parameters. An equilibrium model is employed to match the curves of each corresponding sample. The results are presented in Figure 7 and Table 6, with the dispersion degree (*D_L_ = D/V*) of clay rocks ranging from 0.248 to 0.285 cm.

(2) Advection–dispersion experiments for ^137^Cs/Cs, ^75^Se/Se, and ^99^Tc/Re systems

**Figure 8:**
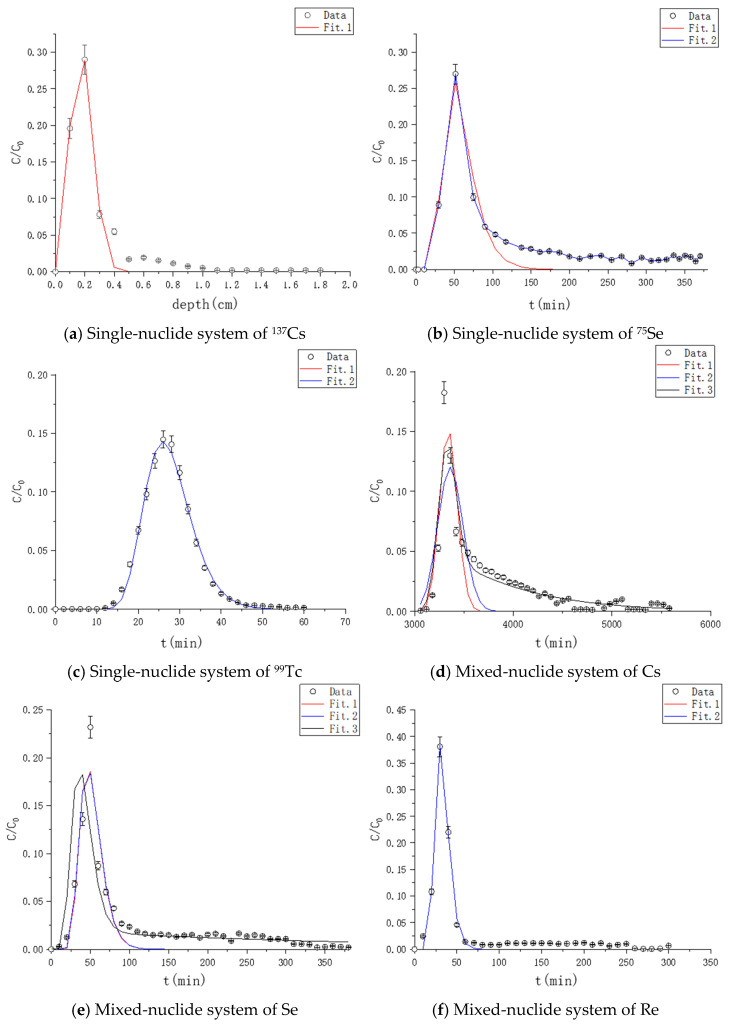
Fitting of advection–dispersion curve (Fit. 1 represents the fitting result of the equilibrium transport model (E-T), Fit. 2 represents the fitting result of the one-site non-equilibrium transport model (O-S), and Fit. 3 represents the fitting result of the two-region non-equilibrium transport model (T-T)). The circular symbols in the figure stand for experimental data, and error bars are used to illustrate the uncertainties arising during the experiments.

Based on the previous adsorption experiment results, it has been observed that competitive adsorption can significantly modify the adsorption mechanism of clay rocks for ^137^Cs and ^79^Se. This effect is further enhanced in advection–dispersion experiments. As shown in Figure 8, the advection–dispersion curves of ^137^Cs and ^79^Se in both single-nuclide and mixed-nuclide systems were fitted using different numerical models. The advection–dispersion curve of ^137^Cs in the single-nuclide system adheres to the equilibrium transport model, whereas the curve for Cs in the mixed-nuclide system necessitates a two-site model for a more accurate fit. This suggests that during the advection–dispersion process, the competitive adsorption effect transforms the initially single strong adsorption site (represented by instantaneous adsorption equilibrium) into a combination of strong adsorption sites (instantaneous adsorption equilibrium) and weak adsorption sites (slower adsorption rate). This phenomenon is also observed for ^79^Se. In the case of ^75^Se, the advection–dispersion curve in a single-nuclide system follows a one-site model (adsorption sites exhibit the same adsorption rate). On the other hand, the curve for Se in a mixed-nuclide system requires a two-site model for improved fitting. This indicates that competitive adsorption similarly changes the original single adsorption site to a combination of strong adsorption sites (instantaneous adsorption equilibrium) and weak adsorption sites (slower adsorption rate).

For the non-adsorbed nuclide ^99^Tc, analysis of the advection–dispersion curve within a single-nuclide system revealed a negative distribution coefficient derived from the equilibrium model. This suggests that ^99^Tc experienced anion repulsion during migration.

The utilization of a two-region model yielded favorable fitting outcomes and determined the moisture content of the non-flowing zone ($\theta_{im}$) and the flowing zone ($\theta_{m}$) due to anion repulsion. By employing both the equilibrium transport model and the two-region model, it was observed that the advection–dispersion curve of Re in the mixed-nuclide system resembled that in the single-nuclide system. However, in the mixed-nuclide system, a two-region model was also necessary to achieve improved fitting outcomes. The distinction lay in the fact that the water content ($\theta_{im}$) in the non-flowing zone was notably lower than that in the single-nuclide system, suggesting that Cs and Se can mitigate the impact of anion repulsion on Re during the competitive adsorption process of “grabbing” adsorption sites.

## 5. Conclusions

**Figure 9:**
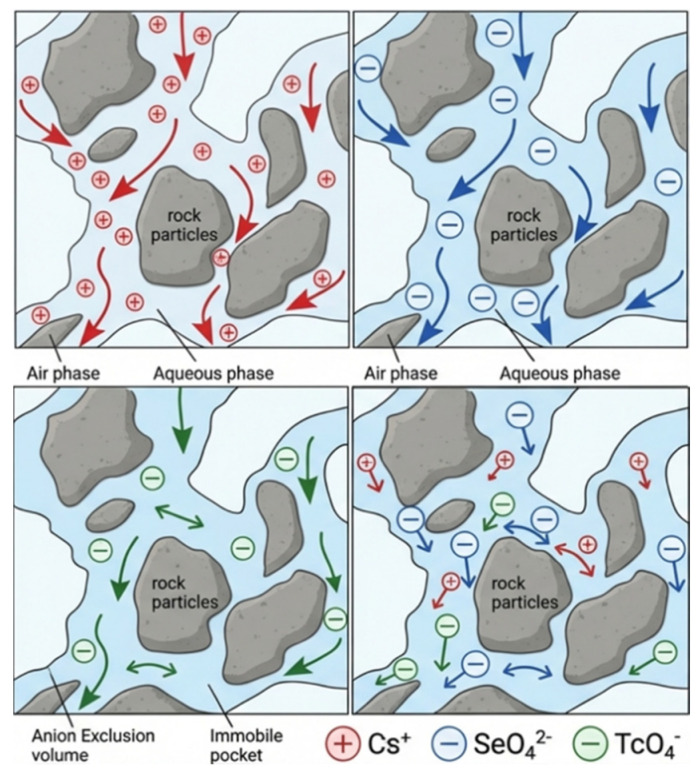
Advection–dispersion mechanism diagram of ^137^Cs, ^79^Se, and ^99^Tc in single-nuclide and mixed-nuclide systems.

The study was performed under specific laboratory conditions and compared batch and column experiments of ^137^Cs, ^79^Se, and ^99^Tc under the coexistence of single- and mixed-nuclide conditions, using different numerical models to fit the experimental data (Figure 9). It further examines changes in adsorption thermodynamics, kinetics, and advection–dispersion behavior of adsorbed nuclides (^137^Cs and ^79^Se) under competitive adsorption. The “anion repulsion” phenomenon exhibited by non-adsorbed nuclides (^99^Tc) during migration is quantitatively characterized by the influence of competitive adsorption. The research conclusions are as follows:

(1) It can be inferred that clay rocks do not exhibit significant adsorption capacity for ^99^Tc. In contrast, competitive adsorption presumably alters the adsorption mechanism of clay rock by competing for robust adsorption sites with two nuclides, notably diminishing the adsorption capacity of clay rocks for ^137^Cs and ^79^Se.
(2) ^137^Cs and ^79^Se were significantly adsorbed during the advection–dispersion process, with competitive adsorption notably reducing the clay rocks’ adsorption capacity for these radionuclides. In particular, ^137^Cs changed from a robust adsorption state of “instantaneous adsorption equilibrium” to two distinct types of adsorption effects, strong and weak, whereas ^79^Se shifted from a singular adsorption point characterized by “the same adsorption rate” to a blend of “fast and slow adsorption rates.”
(3) ^99^Tc experiences significant anion repulsion during the advection–dispersion process. In contrast, ^137^Cs and ^79^Se compete for adsorption sites, potentially reducing the anion repulsion of clay rocks toward ^99^Tc.

## Figures and Tables

**Table 1:** Adsorption experiment condition settings.

Thermodynamic Adsorption Experiment	**Solute State**		**Activity Concentration/Concentration**					**Sampling Time**
			***C*** **_1_**	***C*** **_2_**	***C*** **_3_**	***C*** **_4_**	***C*** **_5_**	
	Single nuclide (Bq∙cm^−3^)	^137^Cs	1000	2000	3000	4000	5000	30 days
		^75^Se	800	1600	2400	3200	4000	
		^99^Tc	800	1600	2400	3200	4000	
	Mixed nuclide (ppm)	Cs	2	5	10	20	50	
		Se	2	5	10	20	50	
		Re	2	5	10	20	50	
Dynamic Adsorption Experiment	**Solute State**		**Sampling Time (day)**					**Solute Content**
			***t*** **_1_**	***t*** **_2_**	***t*** **_3_**	***t*** **_4_**	***t*** **_5_**	
	Single nuclide (Bq∙cm^−3^)	^137^Cs	0.5	3	7	15	30	3000
		^75^Se	0.5	3	7	15	30	2500
		^99^Tc	0.5	3	7	15	30	1500
	Mixed nuclide (ppm)	Cs	0.25	4	7	15	30	10.0
		Se	0.25	4	7	15	30	1.0
		Re	0.25	4	7	15	30	10.0

**Table 2:** Solute input and sampling frequency.

Experiment Type	Solute	Activity/Concentration	Sampling Frequency
HTO column experiment	^3^H	5000 Bq	1 time/3 min
Single-nuclide column experiment	^137^Cs	5000 Bq	1 time/5 min
	^75^Se	3000 Bq	1 time/10 min
	^99^Tc	5000 Bq	1 time/5 min
Mixed-nuclide column experiment	Cs	0.0025 mol∙dm^−3^	1 time/10 min
	Se	0.0026 mol∙dm^−3^	
	Re	0.00081 mol∙dm^−3^	

**Table 3:** Analysis results of mineral composition, chemical composition, and cation exchange capacity.

Mineral Composition (%)	Mica	Quartz	Potassium Feldspar	Plagioclase	Illite	Montmorillonite	Zeolite	Calcite	Dolomite
	11.1	4.0	/	16.9	/	/	23.6	9.6	34.8
Chemical Composition (%)	SiO_2_	Al_2_O_3_	Na_2_O	K_2_O	CaO	Fe_2_O_3_	MgO	TiO_2_	P_2_O_5_
	30.61	9.72	3.08	2.14	27.95	6.74	14.72	0.621	0.080
	SO_3_	MnO	SrO	Rb_2_O	ZrO_2_	ZnO	V_2_O_5_	Cr_2_O_3_	Ga_2_O_3_
	3.74	0.20	0.27	0.02	0.02	0.01	0.02	0.021	0.002
	Co_3_O_4_	Cl	CeO_2_	CuO	Er_2_O_3_	Nd_2_O_3_	ZnO	NiO	Br
	0.003	0.083	0.006	0.005	0.004	0.003	0.014	0.003	0.002
Cation Exchange Capacity				10.936 cmol∙kg^−1^					
Organic Matter Content				27.163 g∙kg^−1^					

**Table 4:** Adsorption isotherm fitting results.

Type	Fit. 1 (L-M)		Fit. 2 (F-M)		Fit. 3 (L-F)	
Single-nuclide system of ^137^Cs	*q_max_*	1.00 × 10^12^ Bq∙g^−1^	*K_f_*	2.19 × 10^1^	/	
	*k_l_*	1.96 × 10^6^ dm^3^∙Bq^−1^	*n*	1.1 × 10^0^		
	*R* ^2^	1.000	*R* ^2^	0.958		
	*RMSE*	0.032	*RMSE*	310.075		
Mixed-nuclide system of Cs	*q_max_*	9.74 × 10^2^ mg∙g^−1^	*K_f_*	1.61 × 10^3^	*q_max_*	6.76 × 10^2^ mg∙g^−1^
					*k_l_*	6.58 × 10^−2^
	*k_l_*	4.75 × 10^−2^ dm^3^∙Bq^−1^	*n*	1.1	*β*	0.84
	*R* ^2^	0.993	*R* ^2^	0.994	*R* ^2^	1.000
	*RMSE*	8.037	*RMSE*	7.660	*RMSE*	4.47
Single-nuclide system of ^79^Se	*q_max_*	1.32 × 10^11^ Bq∙g^−1^	*K_f_*	1.67 × 10^1^	/	
	*k_l_*	1.57 × 10^−10^ dm^3^∙Bq^−1^	*n*	9.52 × 10^−1^		
	*R* ^2^	1.000	*R* ^2^	0.976		
	*RMSE*	0.032	*RMSE*	55.949		
Mixed-nuclide system of Se	*q_max_*	8.25 × 10^4^ mg∙g^−1^	*K_f_*	1.02 × 10^1^	*q_max_*	7.18 × 10^4^ mg∙g^−1^
					*k_l_*	1.50 × 10^−4^
	*k_l_*	1.13 × 10^−4^ dm^3^∙Bq^−1^	*n*	9.85 × 10^−1^	*β*	0.98
	*R* ^2^	0.984	*R* ^2^	0.995	*R* ^2^	1.000
	*RMSE*	23.712	*RMSE*	0.925	*RMSE*	0.152

**Table 5:** Results of adsorption kinetics curve fitting.

Type	Fit. 1 (P-F)		Fit. 2 (P-S)		Fit. 3 (M-S (Second Stage))			
Single-nuclide system of ^137^Cs	*K*_1_	1.010 d^−1^	*K*_2_	0.028 kg∙Bq^−1^∙d^−1^	/			
	*R* ^2^	0.744	*R* ^2^	1.000				
Mixed-nuclide system of Cs	*K*_1_	1.066 d^−1^	*K*_2_	0.340 kg∙Bq^−1^∙d^−1^	*K_f_*_1_	3.290 cm^3^∙(gh)^−1^	*S_m_*_1_	6.42 × 10^2^ mg∙g^−1^
					*K_r_*_1_	0.032 (h^−1^)	*S_m_*_2_	2.31 × 10^1^ mg∙g^−1^
	*R* ^2^	0.801	*R* ^2^	1.000	*K_f_*_2_	0.796 cm^3^∙(gh)^−1^	*K_d_*	101.9 cm^3^∙g^−1^
					*K_r_*_2_	0.036 (h^−1^)	*R* ^2^	1.000
Single-nuclide system of Se	*K*_1_	1.059 d^−1^	*K*_2_	0.002 kg∙Bq^−1^∙d^−1^	/			
	*R* ^2^	0.994	*R* ^2^	1.000				
Mixed-nuclide system of Se	*K*_1_	0.554 d^−1^	*K*_2_	4.33 × 10^4^ kg∙Bq^−1^∙d^−1^	*K_f_*_1_	0.772 cm^3^∙(gh)^−1^	*S_m_*_1_	1.81 × 10^4^ mg∙g^−1^
					*K_r_*_1_	0.089 (h^−1^)	*S_m_*_2_	2.74 × 10^1^ mg∙g^−1^
	*R* ^2^	0.842	*R* ^2^	0.951	*K_f_*_2_	0.081 cm^3^∙(gh)^−1^	*K_d_*	8.65 cm^3^∙g^−1^
					*K_r_*_2_	0.072 (h^−1^)	*R* ^2^	0.999

**Table 6:** Parameters for fitting advection–dispersion curves of various solutes in clay rocks.

Type	Sample-1		Sample-2		Sample-3	
^3^H	*V*	0.383 cm∙min^−1^	*V*	0.358 cm∙min^−1^	*V*	0.322 cm∙min^−1^
	*D*	0.095 cm^2^∙min^−1^	*D*	0.095 cm^2^∙min^−1^	*D*	0.092 cm^2^∙min^−1^
	*D_L_*	0.248 cm	*D_L_*	0.269 cm	*D_L_*	0.285 cm
	*R*	1.00	*R*	1.00	*R*	1.00
	*K_d_*	0	*K_d_*	0	*K_d_*	0
	*RMSE*	5.25 × 10^−6^	*RMSE*	3.89 × 10^−6^	*RMSE*	9.45 × 10^−5^
Type	Fit. 1 (E-T)		Fit. 2 (O-S)		Fit. 3 (T-T)	
Single-nuclide system of ^137^Cs	*V*	0.015 cm∙min^−1^	/		/	
	*D*	0.004 cm^2^∙min^−1^				
	*D_L_*	0.267 cm				
	*R*	9299				
	*K_d_*	1760 mL∙g^−1^				
	*RMSE*	8.62 × 10^−4^				
Mixed-nuclide system of Cs	*V*	0.443 cm∙min^−1^	*V*	0.443 cm∙min^−1^	*V*	0.443 cm∙min^−1^
	*D*	0.0012 cm^2^∙min^−1^	*D*	0.0012 cm^2^∙min^−1^	*D*	0.0012 cm^2^∙min^−1^
	*D_L_*	0.0028 cm	*D_L_*	0.0028 cm	*D_L_*	0.0028 cm
	*R*	148	*a*	0.079	*a*	0.00022
	*K_d_*	27.8 cm^3^∙g^−1^	*K_d_*	28.0 cm^3^∙g^−1^	*K_d_*	31.1 cm^3^∙g^−1^
					*f*	0.889
	*RMSE*	4.14 × 10^−4^	*RMSE*	4.52 × 10^−4^	*RMSE*	1.06 × 10^−4^
Single-nuclide system of ^75^Se	*V*	0.298 cm∙min^−1^	*V*	0.298 cm∙min^−1^	/	
	*D*	0.091 cm^2^∙min^−1^	*D*	0.091 cm^2^∙min^−1^		
	*D_L_*	0.305 cm	*D_L_*	0.305 cm		
	*R*	1.71	*a*	0.029		
	*K_d_*	0.134 cm^3^∙g^−1^	*K_d_*	0.136 cm^3^∙g^−1^		
	*RMSE*	3.62 × 10^−3^	*RMSE*	2.94 × 10^−4^		
Mixed-nuclide system of Se	*V*	0.3 cm∙min^−1^	*V*	0.3 cm∙min^−1^	*V*	0.3 cm∙min^−1^
	*D*	0.116 cm^2^∙min^−1^	*D*	0.116 cm^2^∙min^−1^	*D*	0.116 cm^2^∙min^−1^
	*D_L_*	0.387 cm	*D_L_*	0.387 cm	*D_L_*	0.387 cm
	*R*	1.57	*a*	0.519	*a*	0.00041
	*K_d_*	0.108 cm^3^∙g^−1^	*K_d_*	0.108 cm^3^∙g^−1^	*K_d_*	1.19 cm^3^∙g^−1^
					*f*	0.098
	*RMSE*	2.73 × 10^−4^	*RMSE*	2.65 × 10^−4^	*RMSE*	1.54 × 10^−4^
Type	Fit. 1 (E-T)		Fit. 2 (T-T)		/	
Single-nuclide system of ^99^Tc	*V*	0.256 cm∙min^−1^	*V*	0.256 cm∙min^−1^		
	*D*	0.055 cm^2^∙min^−1^	*D*	0.055 cm^2^∙min^−1^		
	*D_L_*	0.215 cm	*D_L_*	0.215 cm		
	*R*	0.707	$\theta_{m}$	0.16		
	*K_d_*	<0	$\theta_{im}$	0.15		
	*RMSE*	1.60 × 10^−5^	*RMSE*	1.45 × 10^−5^		
Mixed-nuclide system of Re	*V*	0.299 cm∙min^−1^	*V*	0.299 cm∙min^−1^		
	*D*	0.0748 cm^2^∙min^−1^	*D*	0.0748 cm^2^∙min^−1^		
	*D_L_*	0.250 cm	*D_L_*	0.250 cm		
	*R*	0.993	$\theta_{m}$	0.24		
	*K_d_*	<0	$\theta_{im}$	0.07		
	*RMSE*	1.07 × 10^−4^	*RMSE*	1.02 × 10^−4^		

## Data Availability

The original contributions presented in this study are included in the article. Further inquiries can be directed to the corresponding author.
